# Use of the XRCC2 promoter for in vivo cancer diagnosis and therapy

**DOI:** 10.1038/s41419-018-0453-9

**Published:** 2018-03-16

**Authors:** Yu Chen, Zhen Li, Zhu Xu, Huanyin Tang, Wenxuan Guo, Xiaoxiang Sun, Wenjun Zhang, Jian Zhang, Xiaoping Wan, Ying Jiang, Zhiyong Mao

**Affiliations:** 1 Clinical and Translational Research Center of Shanghai First Maternity & Infant Hospital, Shanghai Key Laboratory of Signaling and Disease Research, School of Life Sciences and Technology, Tongji University, 200092 Shanghai, China; 2 Department of Pathophysiology, Key Laboratory of Cell Differentiation and Apoptosis of Ministry of Education, Shanghai Jiao-Tong University School of Medicine, 200025 Shanghai, China

## Abstract

The homologous recombination (HR) pathway is a promising target for cancer therapy as it is frequently upregulated in tumors. One such strategy is to target tumors with cancer-specific, hyperactive promoters of HR genes including RAD51 and RAD51C. However, the promoter size and the delivery method have limited its potential clinical applications. Here we identified the ~2.1 kb promoter of XRCC2, similar to ~6.5 kb RAD51 promoter, as also hyperactivated in cancer cells. We found that XRCC2 expression is upregulated in nearly all types of cancers, to a degree comparable to RAD51 while much higher than RAD51C. Further study demonstrated that XRCC2 promoter is hyperactivated in cancer cell lines, and diphtheria toxin A (DTA) gene driven by XRCC2 promoter specifically eliminates cancer cells. Moreover, lentiviral vectors containing XRCC2 promoter driving firefly luciferase or DTA were created and applied to subcutaneous HeLa xenograft mice. We demonstrated that the pXRCC2-luciferase lentivirus is an effective tool for in vivo cancer visualization. Most importantly, pXRCC2-DTA lentivirus significantly inhibited the growth of HeLa xenografts in comparison to the control group. In summary, our results strongly indicate that virus-mediated delivery of constructs built upon the XRCC2 promoter holds great potential for tumor diagnosis and therapy.

## Introduction

Transcriptional targeting of cancer cells is a mode of gene therapy wherein a cancer-specific promoter drives the selective expression of therapeutic transgenes in order to specifically impede tumor growth with minimal toxicity to normal cells. To date, several tumor-specific promoters have been identified and these promoters may have potential in the diagnosis and treatment of cancer. For instance, caspase-6 transgene expression, driven by the hTERT promoter, whose activity is upregulated in ~90% cancer cells^1^, specifically eliminates glioma cells both in vitro and in vivo^2^. Other cancer-specific promoters employed for transcriptionally targeted cancer therapy include mesothelin^3^, tyrosinase^4^, survivin^5^, midkine^6^, prostate-specific antigen^7^, and human epididymis protein 4^8^. While promising, many of these promoters either do not possess high enough activity to destroy cancer cells or do not exhibit sufficient tissue specificity, posing a threat to non-transformed cells^9,10^. Therefore, a more robustly expressed promoter, which is more narrowly restricted to cancer cells, is needed to better enable the transcriptional targeting of tumor cells.

The homologous recombination (HR) repair pathway is one of the major pathways responsible for repairing DNA double strand breaks in eukaryotes. It safeguards genome integrity in order to prevent the onset of tumorigenesis^11,12^. Cancer cells, however, also take advantage of this pathway to avoid apoptosis induced by the high levels of replication stress associated with their extremely high rates of proliferation. Essential factors such as RAD51 are often overexpressed in a variety of tumor types^13–15^. As a consequence, HR efficiency is frequently upregulated in cancer cells relative to normal cells^16^. Therefore, the aberrantly activated promoters of essential HR genes represent a potential tool for selectively killing cancer cells if they are fused to genes encoding toxic proteins or pro-apoptotic factors. Indeed, the promoters of RAD51 and RAD51C^17,18^, two essential genes involved in HR repair, have been investigated for their potential to transcriptionally target cancer cells. In vitro studies using different types of cancer cells and normal cells indicated that both promoters exhibit strong cancer-specific activity. More intriguingly, in vivo studies using subcutaneous (SC) and intraperitoneal (IP) xenograft models indicated that RAD51 promoter-mediated transcriptional targeting enabled cancer diagnosis and treatment when delivered with nanoparticles^19^. However, although promising, the relatively large size of the RAD51 promoter (~6.5 kb) limits its potential clinical applications as it would greatly impair viral packaging efficiency. As a result, the expression of diagnostic and therapeutic genes may not be high enough to enable tumor cells to be visualized or eliminated. Therefore, for the future clinical applications, it is important to identify a cancer-specific promoter with a relatively small size which is highly activated in a broad array of tumor types. Because RAD51 and RAD51C are highly expressed in some cancer cell types, we hypothesized additional HR factors may also be upregulated in cancer cells and could represent valuable tools for transcriptionally targeting.

XRCC2 is a RAD51 paralog that forms a complex with the other RAD51 paralogs, RAD51B, RAD51C, and RAD51D, to facilitate the step of strand invasion during HR repair^20^. Recent work has indicated that XRCC2 regulates the balance of long-tract and short-tract gene conversions^21^. It has also been well characterized that mutations in XRCC2 are often associated with numerous types of cancers, strongly suggesting that XRCC2 is probably involved in tumorigenesis by regulating HR repair. Indeed, loss of XRCC2 leads to tumorigenesis in brains^22^, indicating that XRCC2 acts as a tumor suppressor in normal tissues. However, whether the XRCC2 promoter is hyperactivated in tumors and its potential use as a tool for the transcriptional targeting of cancer diagnosis and therapy has not been characterized.

Here we found that, similar to RAD51, XRCC2 is upregulated across all types of tumors in comparison to paratumor tissue through data mining of the TCGA database. We further confirmed the upregulation of XRCC2 in cancer cells by comparing the XRCC2 mRNA levels between a collection of cancer cells and normal cells. Moreover, XRCC2 has a smaller promoter that favors its application in transcriptional targeting, compared to RAD51. In vitro studies on the 2101 bp XRCC2 promoter demonstrate that its activity is ~6190-fold higher in cancer cells than in normal cells according to a luciferase reporter assay. The construct comprised of the XRCC2 promoter driving expression of diphtheria toxin A (pXRCC2-DTA) selectively kills cancer cells while having only a very mild effect on normal cells. Most interestingly, using a lentivirus harboring the engineered lentiviral vectors containing pXRCC2-luciferase or pXRCC2-DTA, we found that the lentivirus-mediated delivery of pXRCC2-luciferase can be used as a tool to visualize cancer and that the lentivirus-mediated delivery of pXRCC2-DTA greatly diminished tumor growth in a SC HeLa xenograft model.

## Results

### The mRNA of XRCC2 is upregulated across all types of tumors in comparison to that in paratumors

**Fig. 1:**
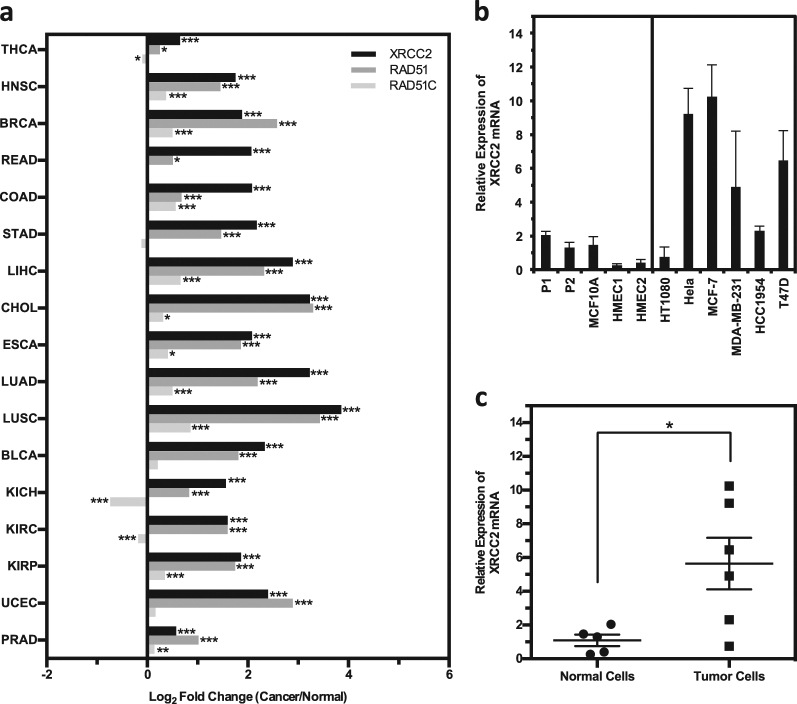
XRCC2 mRNA level is elevated in various types of tumors and cancer cells. **a** RNA-seq data analyzed showed universally high mRNA level in a variety of cancer types. RNA-seq raw count files of 17 cancers, which have paired normal samples, were downloaded from The Cancer Genome Atlas. Differential expression analysis between cancer and normal samples were calculated by the R package DESeq2, which is based on a negative binomial, generalized linear model. The generalized linear models fit returns each gene’s coefficient indicating the log2 fold change. Finally, Wald test was used for statistical analysis (*P_adj_ < 0.05, **P_adj_ < 0.01, ***P_adj_ < 0.001). THCA thyroid cancer, HNSC head and neck squamous cell carcinoma, BRCA breast cancer, READ rectum adenocarcinoma, COAD colon adenocarcinoma, STAD stomach adenocarcinoma, LIHC liver hepatocellular carcinoma, CHOL cholangiocarcinoma, ESCA esophageal carcinoma, LUAD lung adenocarcinoma, LUSC lung squamous cell carcinoma, BLCA bladder carcinoma, KICH kidney chromophobe, KIRC kidney renal clear cell carcinoma, KIRP kidney renal papillary cell carcinoma, UCEC uterine corpus endometrial carcinoma, PRAD prostate adenocarcinoma. **b** Analysis of XRCC2 mRNA level in normal and cancer cells by real-time PCR. GAPDH was used as a reference gene. **c** Quantitative analysis of mRNA expression of XRCC2 in normal and cancer cells. Mann–Whitney *U* test was employed to compare the difference, and the upregulated XRCC2 mRNA level is statistically significant (*P*_MWU_ < 0.05)

Previous reports indicate that the cancer-specific promoters of RAD51 and RAD51C, two critical HR factors, hold great potential for cancer diagnosis and treatment^17,18^, but whether the two promoters are suitable for transcriptionally targeting every type of tumor has not been analyzed. We therefore performed data mining analysis with the TCGA RNA-Seq database, which includes 17 different types of tumors. We found that RAD51 exhibits a robust expression at the mRNA level in all 17 different types of tumors in comparison to paratumor controls (Fig. 1a). In contrast, only 10 out of 17 types of tumors have significantly elevated RAD51C expression than that in paratumor controls. For the remaining seven types of tumors, three of them have significantly reduced RAD51C expression in tumors and no significant difference is observed in the other four types of tumors (Fig. 1a). These results suggest that the RAD51 promoter is potentially more universally activated across all types of tumors than the RAD51C promoter. However, the size of RAD51 promoter is ~6.5 kb which limits its potential clinical applications (Supplementary Figure 1), so expanding the list of cancer-specific promoters would be critical. Intriguingly, we found that XRCC2, another RAD51 paralog, is upregulated at the transcriptional level in all 17 types of tumors compared with that in paratumors. More importantly, the fold change of XRCC2 between tumors and paratumors is even higher in 12 out of 17 types of tumors than that of RAD51 (Fig. 1a), strongly suggesting that the promoter of XRCC2 is another universally hyperactivated promoter in different types of tumors.

To confirm that XRCC2 is highly expressed in cancer cells, we carried out real-time PCR (RT-PCR) to compare XRCC2 mRNA level in several normal cell lines including two human primary fibroblasts (P1-P2), two human mammary epithelial cells (HMEC1 and HMEC2)^17,18^, and a chemically immortalized HMEC cell line, MCF10A, to XRCC2 expression in a panel of cancer cell lines including HeLa, a human cervical carcinoma cell line, HT1080, a human fibrosarcoma cell line, and four human breast cancer cell lines, MCF7, MDA-MB-231, HCC1954, and T47D. Consistent with the data mining result, the average of relative mRNA level of XRCC2 is 5.2-fold higher in cancer cells than that in normal cells (Fig. 1b). Statistical analysis reveals that XRCC2 is significantly higher at mRNA level in cancer cells than that in normal cells (Fig. 1c), indicating that the XRCC2 promoter is potentially cancer specific.

### The XRCC2 promoter is activated in cancer cell lines

**Fig. 2:**
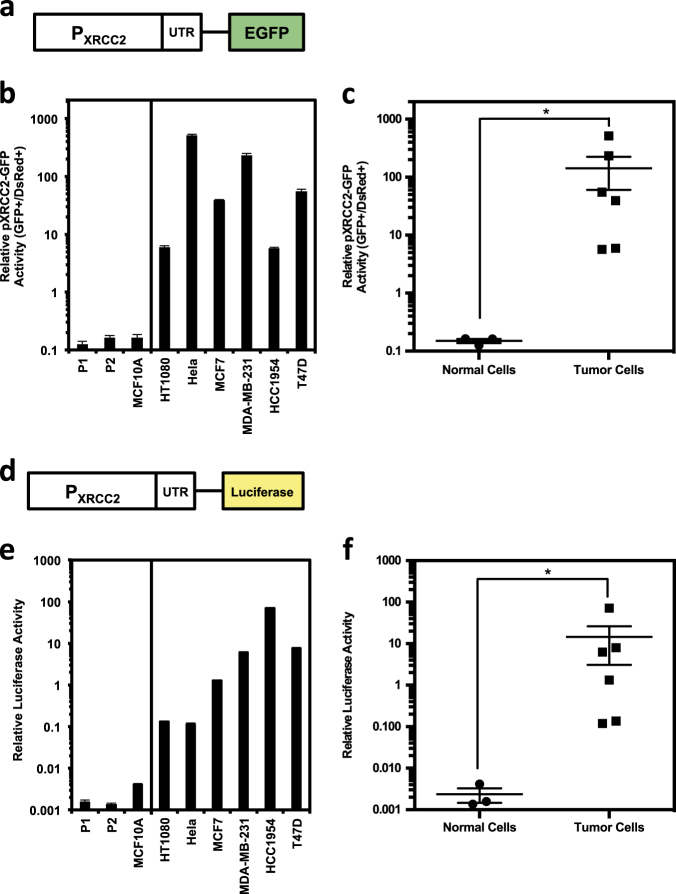
The XRCC2 promoter is hyperactive in cancer cells. **a** Diagram of the pXRCC2-EGFP construct with an EGFP gene driven by the XRCC2 promoter. **b** Analysis of XRCC2 promoter activity in normal and cancer cells. Cells were transfected with pXRCC2-GFP construct and pDsRed-N1. The ratio of GFP+ cells versus DsRed+ cells was used as the measurement of relative promoter activity. **c** Quantitative analysis of XRCC2 promoter activity. Mann–Whitney *U* test indicates that XRCC2 promoter activity is significantly higher in cancer cells than that in normal cells (*P*_MWU_ < 0.05). **d** Diagram of pXRCC2-luciferase construct with firefly luciferase driven by XRCC2 promoter. **e** Analysis of XRCC2 promoter activity in normal and cancer cells. Cells were transfected with pXRCC2-luciferase construct or pEGFP-N1. The ratio of luciferase activity versus the percentage of GFP+ cells was used as the measurement of relative luciferase activity. **f** Quantitative analysis of XRCC2 promoter activity. All experiments were repeated at least three times, and the error bars represent STD. Mann–Whitney *U* test was employed to compare the difference (*P*_MWU_ < 0.05)

To further investigate whether the upregulation of XRCC2 at transcriptional level is a consequence of hyperactivated XRCC2 promoter in cancer cells, we cloned the putative human XRCC2 promoter and its 5′UTR sequence, from −2015 upstream to +86 downstream of the transcription start site (Supplementary Figure 1), into a vector containing an EGFP reporter gene (pXRCC2-EGFP) (Fig. 2a). We then transfected the pXRCC2-EGFP together with pDsRed2-N1 for normalizing transfection efficiency into two normal fibroblasts, MCF10A and the group of cancer cell lines. The ratio of GFP+ cell number versus DsRed+ cell number was employed to compare the XRCC2 promoter activity. We found that the ratio of GFP+/DsRed+ is extremely high in the group of cancer cells in comparison to that in the three normal cell lines (Fig. 2b, c). On average, the XRCC2 promoter activity in cancer cells is ~950-fold higher than that in normal cells (Fig. 2b). The largest difference lies in HeLa cells and P1 fibroblast with a fold change of ~4100 (Fig. 2b). Statistical analysis indicates that the difference between the two groups is significant (Fig. 2c).

Analyzing luciferase activity is another precise way for quantifying promoter activity, so we further cloned the XRCC2 promoter into a firefly luciferase vector (pXRCC2-luciferase) (Fig. 2d). Using the previously described approach^17^, we transfected the reporter vectors and pEGFP-N1 plasmid into exponentially dividing cells respectively. The ratio of firefly luciferase activity versus percentage of GFP+ cells was calculated as the measure of promoter activity. Consistent with the data above, we found that the XRCC2 promoter is significantly hyperactivated in the group of cancer cells (Fig. 2e, f). On average, the XRCC2 promoter activity is 6190-fold higher in the panel of cancer cells than that in three normal cell lines (Fig. 2e), and the largest difference is ~53,000-fold (HCC1954 versus P2 fibroblast), strongly suggesting that the XRCC2 promoter holds great potential for cancer diagnosis and treatment.

### A lentivirus bearing lentiviral pXRCC2-luciferase vector is effective for in vivo imaging of SC tumors

**Fig. 3:**
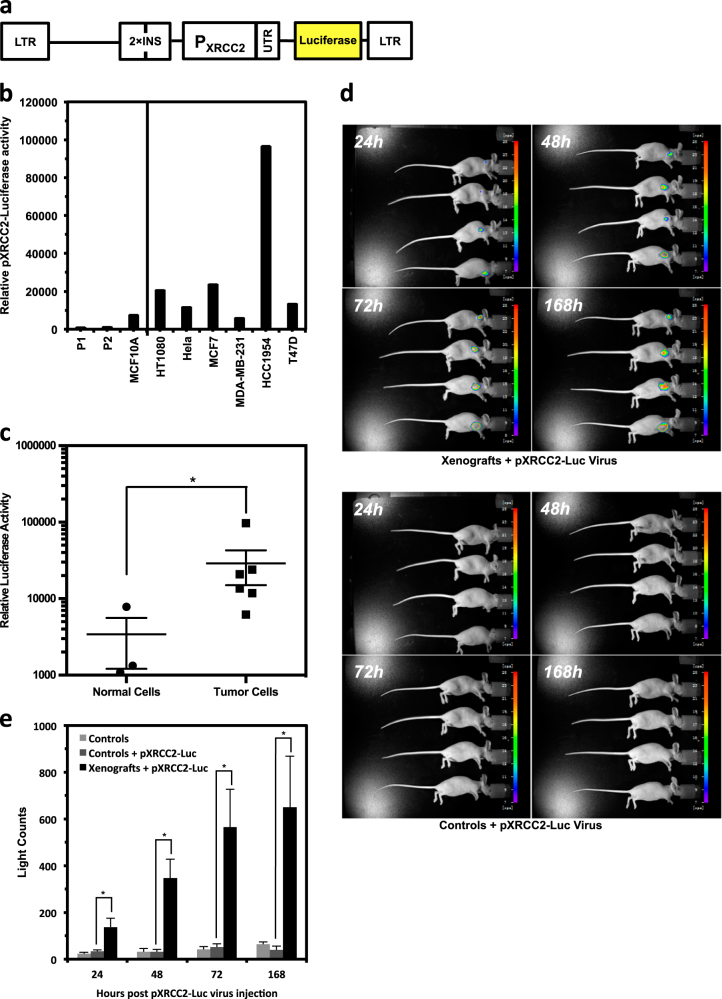
A lentivirus bearing pXRCC2-luciferase is effective for in vivo cancer imaging. **a** Diagram of LTV-pXRCC2-luciferase virus construct. Two insulators were inserted upstream of XRCC2 promoter in order to avoid interferences from CMV and LTR sequences. **b** Luciferase activity was examined on day 3 post pXRCC2-luciferase or GFP lentivirus infections. The ratio of luciferase activity versus the percentage of GFP+ cells was used as the measurement of relative luciferase activity. All experiments were repeated at least three times, and the error bars represent STD. **c** Quantitative analysis of XRCC2 promoter activity. Mann–Whitney *U* test showed that the elevation of XRCC2 promoter activity in cancer cells is statistically significant (*P*_MWU_ < 0.05). **d** Bioluminescent imaging of cancer-free control mouse or xenografted mice injected with pXRCC2-luciferase lentivirus (*n* = 4 for each group). **e** Quantification of the in vivo bioluminescence signal of control or xenograft group. Mann–Whitney *U* test showed that the bioluminescence signal is significantly higher in mice with xenografts than that in control cancer-free mice (24 h, *P* = 0.0316; 48 h, *P* = 0.0085; 72 h, *P* = 0.0194; 168 h, *P* = 0.0319)

To develop the methods of a XRCC2 promoter-based cancer diagnosis and treatment, we first created a lentiviral vector bearing pXRCC2-luciferase based on the FUGW lentiviral vector (Fig. 3a). However, the XRCC2 promoter is heavily influenced by the upstream LTR and CMV promoter, causing a high expression of luciferase independent of XRCC2 promoter in an immortalized normal fibroblast cell line HCA2-hTERT (Figure S1). To minimize the interference, we inserted insulators between the pXRCC2-luciferase and essential viral genes (Supplementary Figure 2a). We found that two insulators greatly diminished the interference in HCA2-hTERT (Supplementary Figure 2b). We then proceeded to package the virus with the lentiviral vector containing pXRCC2-luciferase and insulators, and subsequently infected the groups of normal and cancer cells. We also infected the two groups of cells with a control lentivirus containing FUGW vector for normalizing infection efficiency. We employed the ratio of luciferase versus percentage of GFP+ cells as the measurement of relative XRCC2 promoter activity. We found that although the difference was much milder, possibly due to the remaining interference from upstream LTR and CMV promoter in normal cells, the promoter activity in normal cell lines is significantly lower than that in cancer cell lines (Fig. 3b, c). On an average, the ratio is 8.5-fold lower in normal cell lines than that in cancer cell lines. The largest difference is 88.7-fold (HCC1954 versus P1 fibroblast).

Next, we tested whether the pXRCC2-luciferase virus could be used for in vivo cancer visualization. Tumor-bearing nude mice were established by HeLa cell SC injection. Two weeks later, pXRCC2-luciferase lentivirus (IU = 3.3 × 10^7^) was intratumorally injected twice with a 72-h interval. At 24, 48, 72, and 168 h post the second virus injection, in vivo imaging was carried out to examine luciferase activity. From 24 h post virus injection, bioluminescent signal was detectable in the group of mice with cancer injected with pXRCC2-luciferase virus, while no light counts could be recorded in the group of cancer-free mice with or without viral injection (Fig. 3d, e, Supplementary Figure 3). The bioluminescent signal continued to increase till day 7 post viral injections (Fig. 3d, e).

Our results indicate that pXRCC2-luciferase lentivirus could be potentially applied in clinic for cancer diagnosis with no interference signal from healthy, normal tissues.

### The XRCC2 promoter is effective for transcriptional targeting of cancer therapy in vitro

**Fig. 4:**
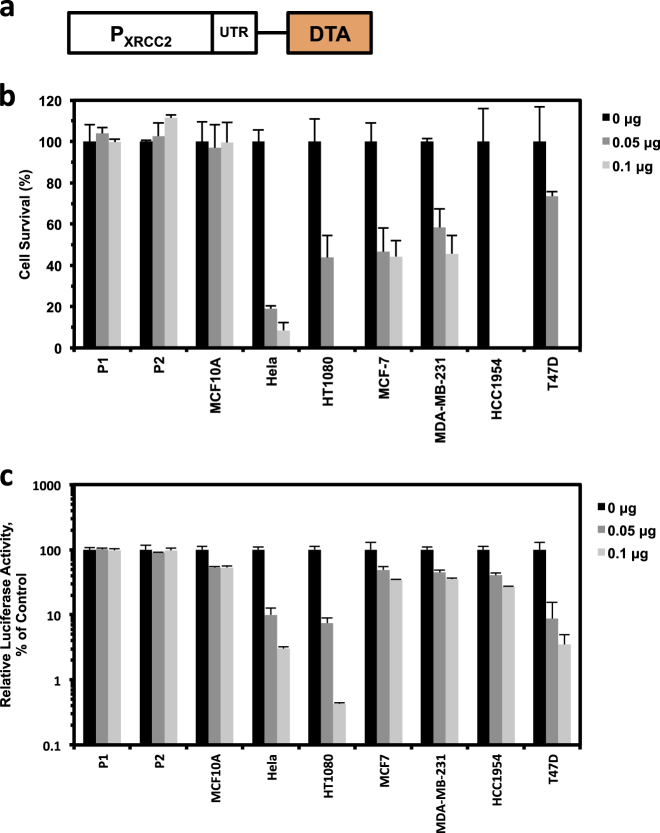
The pXRCC2-DTA vector selectively kills cancer cells in vitro. **a** Diagram of the construct containing DTA gene driven by XRCC2 promoter. **b** Cell survival after pXRCC2-DTA transfection. **c** Inhibition of protein synthesis by pXRCC2-DTA assayed by luciferase analysis. In **b** and **c**, 0, 0.05, or 0.1 μg pXRCC2-DTA, 1 μg SV40-luciferase and pGL3 basic control plasmid to bring the total amount of DNA to 1.1 μg were co-transfected to normal and cancer cells using Fugene 6 reagent. Three days post transfections, cells were harvested, counted, and then luciferase activity was examined. As previously reported^17^, the survival rate of transfected cells (*S*_T_) was calculated using the formula *S*_T_ = *T*_SE_ / *T*_SC_ × 100%, where *T*_SE_ (number of pXRCC2-DTA successfully transfected cell) or *T*_SC_ (number of control plasmid successfully transfected cell) = *H* – *kN*. *H* stands for the total cell number on day 3 post transfections, *k* is the growth rate, and *N* is the number of non-transfected cells (calculated as total cell number used for transfection multiplied by transfection efficiency). All experiments were repeated at least three times

The promoters of RAD51 and RAD51C have been shown to be able to selectively kill cancerous cell lines with minimal effects on normal cell lines once they are fused to DTA, which blocks the process of translation through inactivating eEF2^23^. Similar to RAD51 and RAD51C, the XRCC2 promoter is hyperactivated in the group of cancer cells. We therefore set out to examine whether introduction of a vector containing the XRCC2 promoter driving DTA expression to cells may selectively eliminate cancer cells. After transfecting the two groups of cells with different amounts of plasmid containing DTA gene driven by the XRCC2 promoter (Fig. 4a), we harvested cells and counted the number of cells on a Millipore Muse machine. We found that the pXRCC2-DTA vector may specifically kill all the six cancer cell lines while no significant difference could be observed in the three normal cell lines (Fig. 4b).

To further confirm that the suppressive effect on cancer cell survival was mediated by DTA expression driven by XRCC2 promoter, we performed a previously described assay by co-transfecting pXRCC2-DTA and SV40-luciferase to cells and examining the firefly luciferase activity^17^. In agreement with the results of survival assay, we found that on average the firefly luciferase activity was only mildly inhibited by ~19% and 17.2% with 0.05 μg and 0.1 μg pXRCC2-DTA transfected in normal cells, respectively (Fig. 4c). In contrast, we observed a 73.3% and 82.9% reduction of luciferase activity in the six cancer cell lines (Fig. 4c).

Moreover, we compared the killing effect of pRAD51-DTA and pXRCC2-DTA on HeLa cells. We found that the two promoters exhibit similar inhibitory effect on HeLa cell survival (17.9% versus 23.3%), indicating that the two promoters are probably equally effective on cancer therapy (Supplementary Figure 4).

Our results demonstrate that similar to the previously reported RAD51 and RAD51C promoters, pXRCC2-DTA may also serve as a valuable tool for cancer therapy.

### A lentivirus bearing pXRCC2-DTA significantly impairs the growth of SC xenograft tumors

**Fig. 5:**
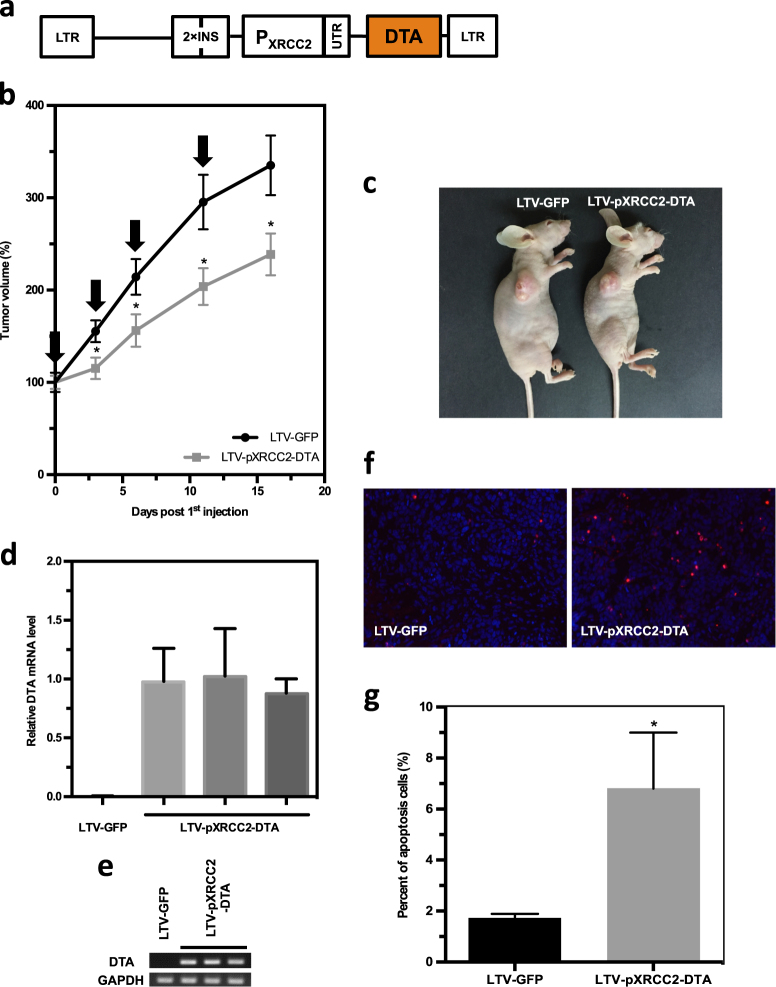
A pXRCC2-DTA lentivirus suppresses tumor growth in vivo. **a** Diagram of LTV-pXRCC2-DTA virus construct. **b** Quantification of tumor volume (*n* = 12 for each group). Arrows indicate the time point of virus injection. Mann–Whitney *U* test showed that the tumor volume of therapy group is significantly smaller than control group (day 3, *P* = 0.0172; day 6, *P* = 0.0283; day 11, *P* = 0.0172; day 16, *P* = 0.0267). **c** Representative image of XRCC2-DTA virus or control virus injected mice with SC HeLa xenografts. The pictures were taken at the end point of the treatment. **d** Analysis of DTA expression at transcriptional level in HeLa xenograft injected with pXRCC2-DTA lentivirus using quantitave PCR. The mRNA was extracted from tissues at 24 h post viral injections, followed by reverse transcription and quantitative analysis of DTA expression. **e** Agarose gel electrophoresis of RT-qPCR products of **d**. **f**, **g** Apoptotic cells in HeLa xenograft injected with pXRCC2-DTA or control viruses were detected using TUNEL assay. Representative TUNEL staining images of different groups. Blue represents DAPI-stained nuclei and red represents TUNEL-positive nuclei **(f)**. Quantification of the TUNEL positive cells **(g)**. The error bars represent STD. *T* test was employed to compare the difference (*P* < 0.05)

To examine the therapeutic efficacy of pXRCC2-DTA in vivo, we created the lentiviral vector containing pXRCC2-DTA by replacing the luciferase gene with DTA on the lentiviral pXRCC2-luciferase plasmid (Fig. 5a).The HeLa xenografts in nude mice were established by inoculating HeLa cells constitutively expressing luciferase on the back of the mice subcutaneously. On day 17 post HeLa cell injection, the packaged virus containing pXRCC2-DTA (IU = 9.3 × 10^5^) was intratumorally injected for the first time, followed by another three injections as indicated (Fig. 5b).

Xenografts were excised for RNA extraction 24 h following the first injection. DTA mRNA could be specifically detected in xenografts receiving pXRCC2-DTA lentiviral treatment (Fig. 5d, e), confirming that DTA was expressed in the tumor tissue.

Each time before injections, the xenografts were measured with calipers. We found that mice receiving pXRCC2-DTA lentivirus treatment had a reduced tumor growth rate in comparison to that in the group receiving control viral injections. At each point of measurement, the average xenograft volume of the therapy group is significantly smaller than that of control group. Five days post the last injection, the average xenograft volume of control group has a 3.4-fold increase compared to the starting point, while only a 2.4-fold increase for therapy group was observed (Fig. 5b, c), suggesting that the virus containing pXRCC2-DTA is an effective tool for attenuating tumor growth rate in vivo.

Previous studies demonstrated that DTA causes the apoptosis of target cells^24^. We therefore performed TUNEL assay on xenograft frozen sections at 48 h post injection. We found that the percentage of TUNEL-positive cells was significantly increased in pXRCC2-DTA lentiviral treatment group in comparison to the control group (Fig. 5f, g), suggesting that DTA-mediated cell apoptosis impairs tumor growth in this model.

## Discussion

A number of tumor types take advantage of HR repair machinery to alleviate replication stress, therefore avoiding apoptosis^13–15^. In contrast, the HR pathway is less active in slowly dividing or quiescent normal cells as it requires the presence of sister chromatids to copy missing information^25^. In addition, the single strand annealing (SSA) pathway, which shares similar repair machinery with HR pathway, is strictly controlled in normal cells to avoid the loss of genetic information between repetitive sequences^26^. Therefore, directly targeting the HR pathway holds the potential to be a promising way to specifically eliminate tumors. Unfortunately, however, a very limited number of small chemical molecules, which directly block HR pathway, have been developed^27^, and more disappointingly none of them have successfully completed clinical development. Therefore, exploring alternative methods of cancer treatment or diagnosis based on the feature of elevated HR pathway in cancer cells is very intriguing.

Our group and others have successfully identified tumor-specific promoters of two HR factors, RAD51 and RAD51C, which can be used for transcriptionally targeting tumor cells in vitro and in vivo. However, although the RAD51 promoter is hyperactive in a panel of cancer cells and it could be used for in vivo imaging and treatment with a type of nanoparticle JetPEI as the delivery method^19^, the toxicity and delivery efficiency of nanoparticles remain the major obstacles to clinical applications^28,29^. Another alternative is to use virus-mediated delivery method, but the promoter size (~6.5 kb) of RAD51 may impair the viral packaging efficiency^29^. In addition, as shown in our data, the mRNA level of RAD51C is not universally high, indicating that RAD51C promoter is probably not ubiquitously hyperactivated across all types of tumors, which may also limit its potential clinical applications. Our newly cloned XRCC2 promoter overcame these two disadvantages. Nearly identical to the size of RAD51C promoter, its size is merely 2.1 kb, which is compatible to most of the viral vectors and does not have a negative effect on viral packaging. In addition, the fold change of XRCC2 mRNA between different types of tumors and their paratumors is similar to that of RAD51 mRNA, indicating that it may be suitable for diagnosing and curing a wide range of tumors.

Although we observed an enormous difference of promoter activity between normal and cancer cells (~6190 fold) on a non-viral vector using electroporation method, we observed a sharp decline of the difference to 8.5-fold when the assay was switched to analyzing promoter activity in cells infected with lentivirus bearing pXRCC2-luciferase. The reason may lie in that although we inserted two insulators to minimize the interference from upstream CMV promoters and LTR, they may still influence transgene expression. Our data suggest that increasing the number of insulators would help overcome this obstacle, but due to the size limitation of the viral vector adding additional insulator would lead to low packaging efficiency, or change the epigenetic landscape of upstream XRCC2 promoter, resulting in promoter silencing. Nevertheless, using the SC HeLa xenograft model, we were still able to distinguish tumor tissues from normal tissues, strongly suggesting that the XRCC2 promoter could be a powerful tool for cancer diagnosis. The next steps would be modifying the lentiviral vector to completely eliminate the interference from other regulatory elements on the XRCC2 promoter, or switching to other viral vehicles such as adenovirus or adenovirus-associated virus (AAV) system in order to improve the cancer specificity. In addition, to avoid the potential toxicity of luciferin used in our research, combining the promoter with reporter genes which can be assayed by widely applied MRI and PET machines in clinic^30,31^ would be another task to accomplish.

Our data demonstrated that using lentivirus-mediated delivery of pXRCC2-DTA significantly suppressed the growth of tumors in vivo, strongly indicating that utilization of the XRCC2 promoter for cancer therapy with a viral delivery is extremely promising. This newly developed tool holds the potential of treating a variety of tumors as it is hyperactivated in nearly all types of tumors tested. However, the lentiviral vector may transform normal cells by integrating into genomes of normal cells and causing aberrant gene expressions. To solve this problem, we may employ non-integrative adenovirus or AAV as the means for delivery. Another potential risk of this therapeutic method is the unwanted expression of pXRCC2-driven expression of DTA in different types of normal tissues, particularly in somatic stem cells which may have active HR repair due to its potential to replenish damaged cells to maintain tissue homeostasis^32,33^. To avoid this risk, we may perform genetic engineering on the viral glycoproteins to improve its specificity for tumor tissues^34^. Another way of mitigating this concern is that we may modify the virus envelope protein in order to promote its affinity to Fc fragment of immunoglobulins. By incubating the modified virus with monoclonal antibody recognizing the cellular surface antigen of target cells, we could also achieve the goal of improving specificity of infecting tumor tissues^35,36^.

How to reconcile the controversy that the change of XRCC2 mRNA between paratumors and tumors, or between normal and cancer cells, is not as dramatic as the change of XRCC2 promoter activity assayed by exogenous reporter genes? We hypothesize that similar to cloned RAD51 and RAD51C promoters, the suppressive regulatory DNA elements of XRCC2 are not included in the 2.1 kb promoter. In addition, the regulation of XRCC2 mRNA stability by splicing or microRNA or other post-transcriptional mechanisms may also lead to the difference we observed. Nevertheless, a thorough study on the regulation of the cancer-specific activation of these promoters is needed.

In summary, the newly identified XRCC2 promoter and its utilization in the transcriptional targeting of cancer cells could achieve the major goals of cancer therapy: high selectivity and efficacy. Although the way of virus-mediated delivery needs to be further improved, our proof-of-concept study demonstrates the feasibility of using viral vehicles containing XRCC2 promoter for cancer diagnosis and treatment in various types of tumors in vivo.

## Materials and methods

### Cell culture and transfection

Two primary human fibroblasts were isolated from the skin of abdomen (P1) and eyelid (P2) of two healthy donors of age 33 and 24 years. The human subjects used in this research are in accordance with the Ethic Committees of Tongji University. Protocol of fibroblast isolation from human tissues is as previously reported^37,38^. Fibroblasts were cultured in MEM medium supplemented with 10% FBS, 1% Penicillin/Streptomycin, and 1% NEAA. MCF10A was cultured in Lonza MEGM medium. HeLa, HT1080, MCF7, and T47D were cultured in DMEM medium with 10% FBS and 1% Penicillin/Streptomycin. MDA-MB-231 was cultured in L-15 medium with 10% FBS and 1% Penicillin/Streptomycin. HCC1954 was kept in 1640 medium with 10% FBS and 1% Penicillin/Streptomycin. All cell lines were maintained at 37 °C, 3% O_2_, and 5% CO_2_.

For GFP and luciferase transfection, all cells were collected and transfected with 0.5 μg plasmid at 48 h post splitting using a Lonza 4D electroporation machine (Lonza, Germany) with the following programs: human primary fibroblasts, DT130; MCF10A, EL110; HeLa, CN114; HT1080, FF113; MCF-7, CM130; HCC1954 and T47D, FF150; MDA-MB-231, FF138. Cells were harvested for FACS analysis 48 h post transfection and analyzed on FACSverse (BD Biosciences, USA). Further analysis was performed with Flowjo software. For DTA killing and luciferase inhibition assay, cells were transfected with 1 μg pSV40-Luciferase and 0, 0.05, or 0.1 μg pXRCC2-DTA plasmid supplemented with pGL3 basic plasmid to bring the total amount of DNA to 1.1 μg using Fugene 6 transfection reagent (Roche) as previously described. Cells were harvested and counted at 72 h post transfection before analysis of luciferase activity.

### RT-PCR

Fifty nanograms of cDNA or genomic DNA was used as template for RT-PCR using FastStart Universal SYBR Green Master (ROX) (Roche). RT-PCR was performed on an ABI7500 RT-PCR machine (Life Technologies, CA). Primers for quantifying XRCC2 mRNA level are as follows: 5′: GCAGTTGGTGAATGGCGTTG and 3′: TTCAAGGAACTTCTACCTTC. GAPDH was used as a reference gene. Primers for titrating virus are as follows: 5′: AGGAGCTTTGTTCCTTGGGT and 3′: AGGAGCTGTTGATCCTTTAG. Primers for quantifying DTA mRNA level are as follows: 5′: GGTTCGGTGATGGTGCTTCG and 3′: CACGATTTCCTGCACAGGCT.

### Generation of pXRCC2-EGFP, pXRCC2-luciferase, pXRCC2-DTA, and lentiviral vectors containing pXRCC2-luciferase, pXRCC2-DTA

The XRCC2 promoter was amplified from genomic DNA isolated from HCA2 cells using primers 5′: TTCGAATTCGCGCTTCAAGTATCCTTTTAAACGAG and 3′: GAGACCGGTCGCCCCGAAGGCTCGGCGCAGGA, and then subcloned into the pRAD51C-GFP plasmid by replacing the RAD51C promoter with AgeI and EcoRI. Using similar approach, to create pXRCC2-luciferase, XRCC2 promoter was cloned into pGL3 plasmid using NheI and HindIII. For the pXRCC2-DTA vector, the DTA ORF was amplified with primers 5′: GGCACCGGTGCCACCATGGATCCTGATGATGTTGTTATTC and 3′: GTCGCGGCCGCTTAGAGCTTTAAATCTCTGTAGG, followed by replacing EGFP gene with DTA gene on the pXRCC2-EGFP vector.

For the two lentiviral vectors containing pXRCC2-luciferase or pXRCC2-DTA, we first modified the FUGW vector by inserting a DNA oligo5′: TAAGCTAGCTCTAGAG and 3′: AATTCTCTAGAGCTAGCTTAAT at the restriction enzyme site PacI and EcoRI. Then the pXRCC2-luciferase was taken from pXRCC2-Luciferase vector with NheI and XbaI enzymes, and the fragment was utilized to replace UBC-EGFP on the FUGW vector (FUW-pXRCC2-luciferase). Then, one or two insulators were subcloned into FUW-pXRCC2-luciferase to generate lentiviral vector containing pXRCC2-luciferase (LTV-pXRCC2-luciferase). Primers for insulator amplifying are 5′: GTTTAAACGCTAGCGGCGCGCCGGC and 3′: TCGGCTAGCTTAATTAAGGATCCCCGGGTACCAAT and the insulators are on the vector of pNEB_Ins TRE-Tight I-SceI. For LTV-pXRCC2-DTA vector, pXRCC2-DTA were first cloned into oligo-inserted FUGW vector using NheI and XbaI, resulting in FUGW-pXRCC2-DTA. Then, pXRCC2-DTA was digested with NheI and ligated into LTV-pXRCC2-Luciferase to replace pXRCC2-Luciferase, generating final LTV-pXRCC2-DTA.

### Luciferase assay

Cells were collected and counted at 72 h post transfection. Cells were then lysed using passive lysis buffer (Promega, Cat. # E1491) at a volume of 200 μL for every 10^6^ cells. Ten microliters cell lysate was mixed with 50 μL firefly luciferase substrate (Promega, Cat. # E1960) for luciferase activity test on a GloMax20/20 luminometer (Promega, USA). To normalize the difference of transfection efficiency, cells were transfected with 5 μg pCMV-GFP and analyzed by FACS. Relative luciferase activity was calculated as the ratio of luciferase activity versus percentage of GFP+ cells.

### Bioinformatic analysis

HTSeq raw count files of 17 cancers which have paired normal samples were downloaded from The Cancer Genome Atlas. Fold changes were calculated by DESeq2^39^.

### Virus packaging

At 24 h post seeding, HEK293FT cells were transfected with 2.5 μg VSVG, 3.75 μg delta 8.9, and 5 μg LTV-pXRCC2-Luciferase/DTA, using p-PEI reagent. At 72 h post transfection, the supernatant was collected, filtered, and centrifuged at a speed of 55,000×*g* on an ultra-speed centrifuge (Beckman Coulter). The precipitation was re-suspended with DMEM media, aliquoted and stored at −80 °C before intratumoral injections or directly used for viral titration.

### Xenografts and viral injection

Eight-week-old nude mice were used for xenograft tumor formation. For xenograft, 200 μL solution containing 5 × 10^5^ Hela cells and 20% matrigel in PBS were injected subcutaneously into the foreleg/upper back region of mice. The xenografts were allowed to grow for 2 weeks. For tumor visualization, pXRCC2-luciferase lentivirus (IU = 3.3 × 10^7^ each time) was intratumorally injected twice into cancer/cancer-free mice with a 72-h interval. In vivo luciferase activity was measured at 24 h, 48 h, 72 h, and 1 week after the second injection.

For tumor treatment, xenografts were allowed to grow for 2.5 weeks before the first pXRCC2-DTA lentivirus injection. Tumor size was measured with calipers each time before injection. Mice received a total of four intratumoral injections of pXRCC2-DTA lentivirus for therapy group and GFP lentivirus for control group at the titer of 9.3 × 10^5^ infection unit virus. The therapy lasted for 11 days until large ulceration occurred in the tumors of most control mice, and all procedures involving animals were approved by the Laboratory Animal Care Committee of Tongji University.

### TUNEL assay

At 48 h post injection, xenografts were excised and fixed with 4% paraformaldehyde at 4 °C overnight, followed by cryoprotection with 30% sucrose and O.C.T embedding. TUNEL assay was performed following the detailed procedure described in In Situ Cell Death Detection Kit, TMR red (Roche, Cat. # 12156792910).

## Electronic supplementary material

Supplementary Figure Legends(DOCX 12 kb)

Supplementary Figure 1–4(PDF 459 kb)

## References

[1] Shay JW, Bacchetti S (1997). A survey of telomerase activity in human cancer. Eur. J. Cancer.

[2] Komata T (2001). Treatment of malignant glioma cells with the transfer of constitutively active caspase-6 using the human telomerase catalytic subunit (human telomerase reverse transcriptase) gene promoter. Cancer Res..

[3] Breidenbach M (2005). Mesothelin-mediated targeting of adenoviral vectors for ovarian cancer gene therapy. Gene Ther..

[4] Nettelbeck DM, Rivera AA, Balague C, Alemany R, Curiel DT (2002). Novel oncolytic adenoviruses targeted to melanoma: specific viral replication and cytolysis by expression of E1A mutants from the tyrosinase enhancer/promoter. Cancer Res..

[5] Chen JS (2004). Cancer-specific activation of the survivin promoter and its potential use in gene therapy. Cancer Gene Ther..

[6] Yu L (2004). Midkine promoter-driven suicide gene expression and -mediated adenovirus replication produced cytotoxic effects to immortalised and tumour cells. Eur. J. Cancer.

[7] Latham JP, Searle PF, Mautner V, James ND (2000). Prostate-specific antigen promoter/enhancer driven gene therapy for prostate cancer: construction and testing of a tissue-specific adenovirus vector. Cancer Res..

[8] Rawlinson JW, Vaden K, Hunsaker J, Miller DF, Nephew KP (2013). Adenoviral-delivered HE4-HSV-tk sensitizes ovarian cancer cells to ganciclovir. Gene Ther. Mol. Biol..

[9] Lo HW, Day CP, Hung MC (2005). Cancer-specific gene therapy. Adv. Genet..

[10] Bilsland AE, Fletcher-Monaghan A, Keith WN (2005). Properties of a telomerase-specific Cre/Lox switch for transcriptionally targeted cancer gene therapy. Neoplasia.

[11] Khanna KK, Jackson SP (2001). DNA double-strand breaks: signaling, repair and the cancer connection. Nat. Genet..

[12] Hsu HM (2007). Breast cancer risk is associated with the genes encoding the DNA double-strand break repair Mre11/Rad50/Nbs1 complex. Cancer Epidemiol. Biomarkers Prev..

[13] Raderschall E (2002). Elevated levels of Rad51 recombination protein in tumor cells. Cancer Res..

[14] Xia SJ, Shammas MA, Shmookler Reis RJ (1997). Elevated recombination in immortal human cells is mediated by HsRAD51 recombinase. Mol. Cell. Biol..

[15] Maacke H (2000). DNA repair and recombination factor Rad51 is over-expressed in human pancreatic adenocarcinoma. Oncogene.

[16] Mao Z, Jiang Y, Liu X, Seluanov A, Gorbunova V (2009). DNA repair by homologous recombination, but not by nonhomologous end joining, is elevated in breast cancer cells. Neoplasia.

[17] Hine CM, Seluanov A, Gorbunova V (2008). Use of the Rad51 promoter for targeted anti-cancer therapy. Proc. Natl Acad. Sci. USA.

[18] Cao Y (2014). Utilization of Rad51C promoter for transcriptional targeting of cancer cells. Oncotarget.

[19] Hine CM, Seluanov A, Gorbunova V (2012). Rad51 promoter-targeted gene therapy is effective for in vivo visualization and treatment of cancer. Mol. Ther..

[20] Shim KS, Schmutte C, Tombline G, Heinen CD, Fishel R (2004). hXRCC2 enhances ADP/ATP processing and strand exchange by hRAD51. J. Biol. Chem..

[21] Nagaraju G, Hartlerode A, Kwok A, Chandramouly G, Scully R (2009). XRCC2 and XRCC3 regulate the balance between short- and long-tract gene conversions between sister chromatids. Mol. Cell. Biol..

[22] Frappart PO (2009). Recurrent genomic alterations characterize medulloblastoma arising from DNA double-strand break repair deficiency. Proc. Natl Acad. Sci. USA.

[23] Foley BT, Moehring JM, Moehring TJ (1995). Mutations in the elongation factor 2 gene which confer resistance to diphtheria toxin and Pseudomonas exotoxin A. Genetic and biochemical analyses. J. Biol. Chem..

[24] Chang MP (1989). Internucleosomal DNA cleavage precedes diphtheria toxin-induced cytolysis. Evidence that cell lysis is not a simple consequence of translation inhibition. J. Biol. Chem..

[25] Jazayeri A (2006). ATM- and cell cycle-dependent regulation of ATR in response to DNA double-strand breaks. Nat. Cell Biol..

[26] Mao Z, Bozzella M, Seluanov A, Gorbunova V (2008). Comparison of nonhomologous end joining and homologous recombination in human cells. DNA Repair.

[27] Chernikova SB, Game JC, Brown JM (2012). Inhibiting homologous recombination for cancer therapy. Cancer Biol. Ther..

[28] Yin H (2014). Non-viral vectors for gene-based therapy. Nat. Rev. Genet..

[29] Yin H, Kauffman KJ, Anderson DG (2017). Delivery technologies for genome editing. Nat. Rev. Drug Discov..

[30] Amer, M. H. Gene therapy for cancer: present status and future perspective. *Mol. Cell. Ther.* **2**, 27 (2014). 10.1186/2052-8426-2-27 PMC4452068 26056594

[31] Wu L, Johnson M, Sato M (2003). Transcriptionally targeted gene therapy to detect and treat cancer. Trends Mol. Med..

[32] Biteau B, Hochmuth CE, Jasper H (2011). Maintaining tissue homeostasis: dynamic control of somatic stem cell activity. Cell Stem Cell.

[33] Fuchs E, Chen T (2013). A matter of life and death: self-renewal in stem cells. EMBO Rep..

[34] Escors D, Breckpot K (2010). Lentiviral vectors in gene therapy: their current status and future potential. Arch. Immunol. Ther. Exp. (Warsz).

[35] Morizono K, Bristol G, Xie YM, Kung SK, Chen IS (2001). Antibody-directed targeting of retroviral vectors via cell surface antigens. J. Virol..

[36] Zhang KX (2009). Lentiviruses with trastuzumab bound to their envelopes can target and kill prostate cancer cells. CancerGene Ther..

[37] Seluanov A (2008). Distinct tumor suppressor mechanisms evolve in rodent species that differ in size and lifespan. Aging Cell.

[38] Xu Z (2015). SIRT6 rescues the age related decline in base excision repair in a PARP1-dependent manner. Cell Cycle.

[39] Love MI, Huber W, Anders S (2014). Moderated estimation of fold change and dispersion for RNA-seq data with DESeq2. Genome Biol..

