# Supplementary material for: Use of the XRCC2 promoter for in vivo cancer diagnosis and therapy
Source: Cell Death Dis. 2018 Mar 16;9(4):420. doi: 10.1038/s41419-018-0453-9 (PMC5856804; doi:10.1038/s41419-018-0453-9)
Supplement: Supplementary file 1 — Supplementary Figure Legends(DOCX 12 kb) [file 41419_2018_453_MOESM1_ESM.docx]

**Supplementary Figure Legends**

**Supplementary Figure 1.** Comparison of the size of RAD51, RAD51C and XRCC2 promoter. **(a)** The size of XRCC2 promoter is 3-fold smaller than the functionally similar RAD51 promoter. **(b)** Diagram of XRCC2 promoter. CCAAT box and TATA box are predicted by EPD (Eukaryotyic promoter database), potential transcription factor binding sites are predicted by PROMO.

**Supplementary Figure 2.** Insulators block the interference from CMV and LTR elements when analyzing the luciferase activity. **(a)** Diagram of LTV-pXRCC2-Luciferase vector with none, 1 or 2 insulators. **(b)** Luciferase activity was examined 3 days post 0.5 μg vectors transfected into HCA2-hTERT cells.

**Supplementary Figure 3.** The bioluminescent image of control cancer-free mice with no pXRCC2-luciferase virus injection.

**Supplementary Figure 4.** XRCC2 promoter exhibits comparably inhibitory effect on HeLa cell survival to RAD51 promoter. Luciferase activity was examined 3 days post 0 or 0.1 μg pXRCC2-DTA vector transfected into HeLa cells.
