# Supplementary figures and images for: Use of the XRCC2 promoter for in vivo cancer diagnosis and therapy
Source: Cell Death Dis. 2018 Mar 16;9(4):420. doi: 10.1038/s41419-018-0453-9 (PMC5856804; doi:10.1038/s41419-018-0453-9)

**a**

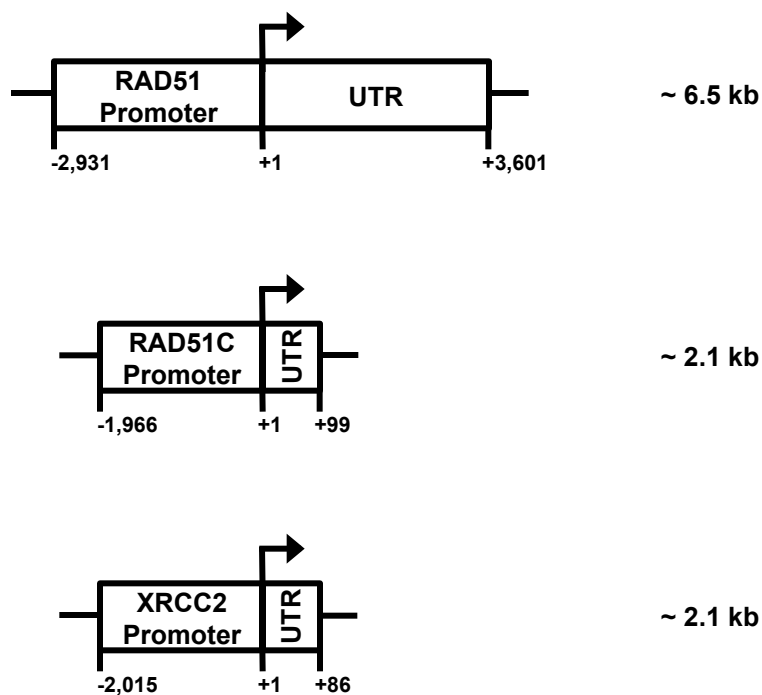

**b**

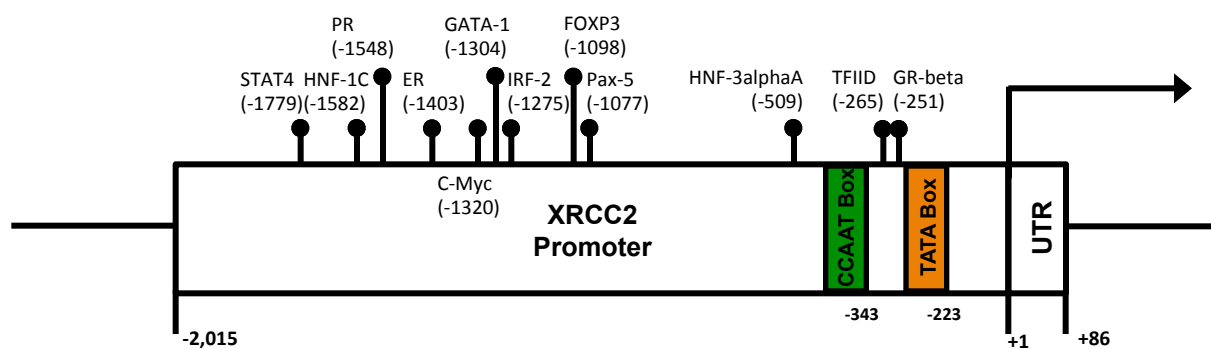

**a**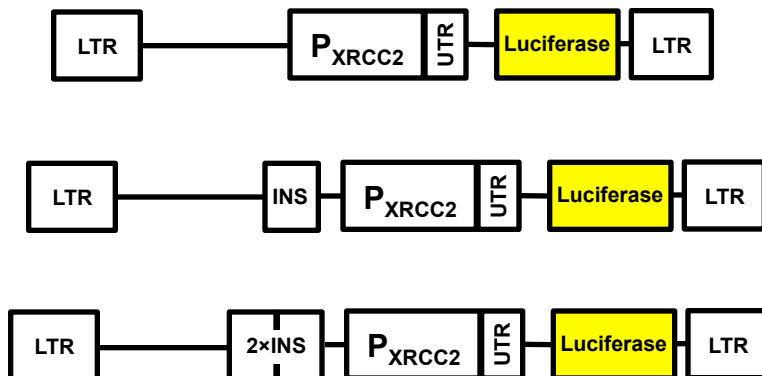**b**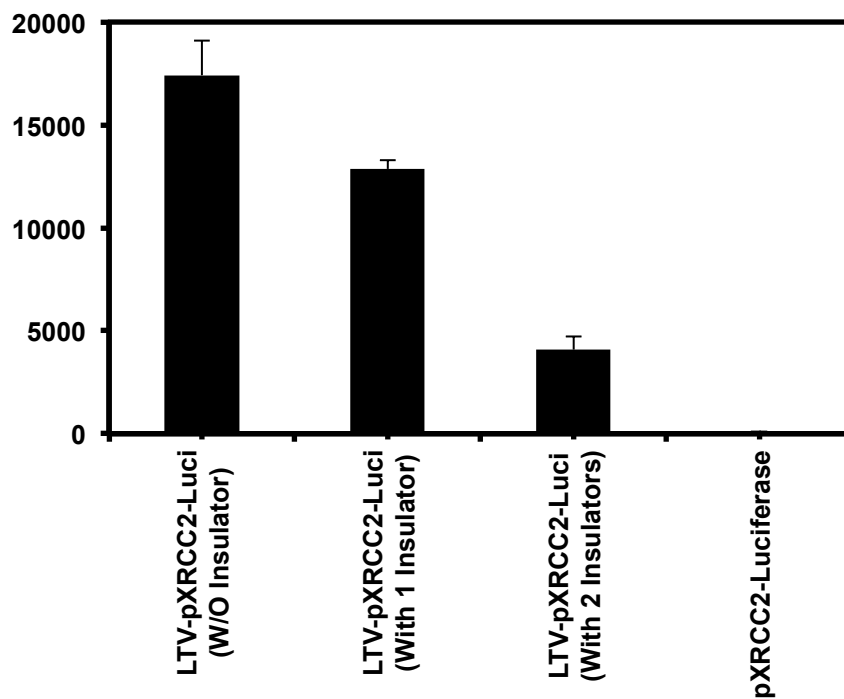**Supplementary Figure 2**

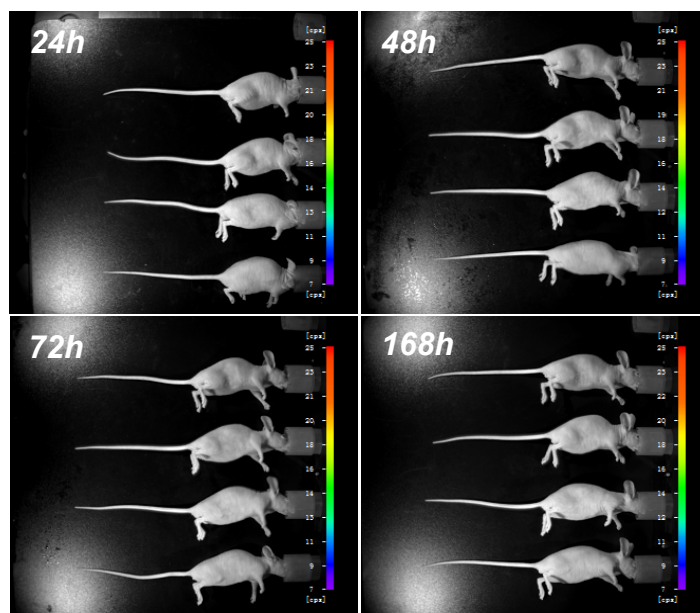

Controls without pXRCC2-Luc Virus

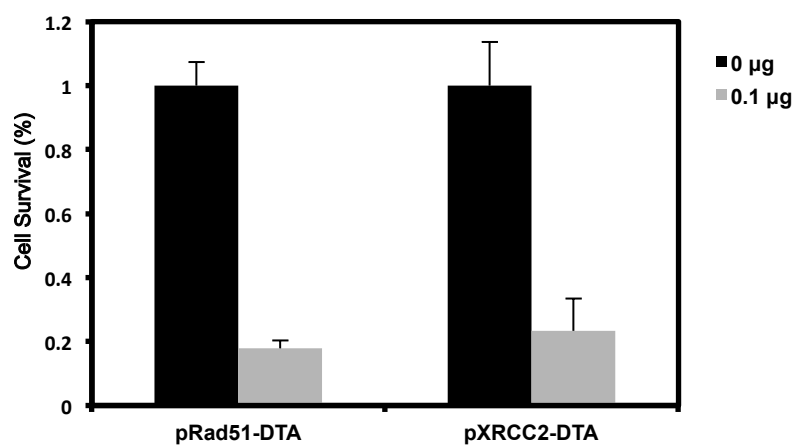

**Supplementary Figure 4**

Supplement: Supplementary file 2 — Supplementary Figure 1–4(PDF 459 kb) [file 41419_2018_453_MOESM2_ESM.pdf]
